# Association between Sjögren’s Syndrome and Periodontitis: Epidemiological, Fundamental and Clinical Data: A Systematic Review

**DOI:** 10.3390/diagnostics13081401

**Published:** 2023-04-12

**Authors:** Dorin Nicolae Gheorghe, Dora Maria Popescu, Stefan Cristian Dinescu, Margarita Silaghi, Petra Surlin, Paulina Lucia Ciurea

**Affiliations:** 1 Doctoral School, University of Medicine and Pharmacy of Craiova, 200349 Craiova, Romania; 2 Department of Periodontology, Research Center of Periodontal-Systemic Interactions, Faculty of Dental Medicine, University of Medicine and Pharmacy of Craiova, 200349 Craiova, Romania; popescu131@yahoo.com (D.M.P.); surlinpetra@gmail.com (P.S.); 3 Department of Internal Medicine—Rheumatology, Faculty of Medicine, University of Medicine and Pharmacy of Craiova, 200349 Craiova, Romania; ciureapaulina@yahoo.com; 4 Faculty of Dental Medicine, University of Medicine and Pharmacy of Craiova, 200349 Craiova, Romania; margaritasil@outlook.com.gr

**Keywords:** Sjögren’s syndrome, periodontitis, periodontal disease, pathogenesis, risk factor, status

## Abstract

In recent decades, researchers have investigated the bidirectional links between periodontal disease and systemic diseases, and the results have allowed the development of the concept of periodontal medicine. This concept incorporates and analyzes the mutually influential interactions that can occur between periodontitis and systemic diseases such as diabetes mellitus or cardiovascular diseases. Sjögren’s syndrome (SS) is a chronic autoimmune disorder that targets the exocrine glands of the body, such as the lacrimal and salivary glands. The amount of saliva produced may gradually decrease with the progression of the disease, which can have an impact on the structures within the oral cavity. Although the reduction in saliva flow produces negative effects in the oral cavity, a direct association between Sjögren’s syndrome and periodontal disease has not yet been demonstrated. Available studies on this topic have not identified significant differences in the periodontal status of patients with Sjögren’s syndrome and control groups at the clinical and bacteriological levels. On the other hand, other studies on this topic consider that patients with periodontitis have a higher risk of developing Sjögren’s syndrome than the general population. Therefore, the results remain inconclusive, highlighting the need for further complementary studies.

## 1. Introduction

Periodontitis is a chronic inflammatory illness that affects the periodontal tissue, which includes the teeth’s supporting structures such as the gums, alveolar bone, cementum, and periodontal ligament [1]. It is distinguished by the breakdown of these tissues, which leads to tooth loss if untreated [1]. Periodontitis has a complex etiology; however, it is predominantly caused by bacterial plaque and its metabolites. The bacteria colonize the tooth surfaces and form a biofilm, which triggers the host’s immunological response [2]. The host reaction to the bacterial plaque triggers the production of inflammatory mediators, which can contribute to periodontal damage when exceeding a threshold [3]. Periodontitis signs include clinical attachment loss, gingival recession, and alveolar bone loss [4]. It is a serious public health problem because it causes considerable morbidity and is the main cause of adult tooth loss, but early detection of its symptoms (such as gingival redness, swelling and bleeding, or bad breath and painful mastication) could prevent its expansion and development of significant clinical consequences, through adequate treatment [4]. Periodontitis therapy involves mechanical plaque reduction, removal of local irritants, and the use of antibacterial medicines [4].

Periodontal medicine is a branch of periodontology that focuses on the relationship between periodontal disease and overall health [5]. This field of study seeks to comprehend the pathophysiology of periodontal disease, the vulnerability of the host to the illness, and the impact of periodontal disease on general health. It entails combining classical periodontology, medicine, and other pertinent sciences in order to provide holistic patient care [5].

Periodontal medicine practitioners employ a multidisciplinary approach in the management of patients with periodontal disease, collaborating with other healthcare professionals to provide coordinated care [6]. They are responsible for the identification, diagnosis, treatment, and management of patients at increased risk for periodontal disease, such as those with systemic conditions, including diabetes, cardiovascular disease, and pregnancy, and those with genetic predisposition or environmental risk factors, such as tobacco use [7]. The ultimate goal of periodontal medicine is to improve the overall health of patients by addressing periodontal disease, and to enhance the understanding of how periodontal disease may affect systemic health, which will facilitate the development of new treatment and preventive strategies [8].

Sjögren’s syndrome (SS) (Abbreviations) is a chronic autoimmune disorder characterized by lymphocytic infiltration of exocrine glands, particularly the lacrimal and salivary glands, leading to a decreased production of tears and saliva (sicca syndrome) [9]. The exact etiology of Sjögren’s syndrome is not completely understood; however, it is believed to be a complex interaction between genetic and environmental factors. Women are more frequently affected than men, with a female-to-male ratio of 9:1 [9,10].

Sjögren’s syndrome is a multi-system disorder that mainly affects the salivary and lacrimal glands, with potential involvement of the exocrine glands of the gastrointestinal tract, as well as various organs such as the lungs, kidneys, liver, joints, and the central nervous system [11,12]. Additionally, it can be associated with other autoimmune disorders such as rheumatoid arthritis, systemic lupus erythematosus, and scleroderma [13]. There is no cure for Sjögren’s syndrome; management of the disease is symptomatic and includes the use of artificial tears and saliva substitutes [14]. In some cases, immunosuppressive drugs may be used to control the disease [14]. Early diagnosis and treatment can help prevent complications and improve the quality of life for patients with Sjögren’s syndrome [15].

The disorder can be classified into two main categories: primary and secondary [16]. Primary Sjögren’s syndrome (pSS) is defined as a stand-alone disorder in which the exocrine glands are primarily affected, and it is not associated with any other underlying autoimmune disorder. The diagnosis of primary Sjögren’s syndrome is established by fulfilling the criteria set by the American–European Consensus Group (AECG), which includes the clinical features of xerophthalmia and xerostomia along with the presence of anti-Sjögren’s syndrome-related antigen A autoantibodies (anti-SSA/Ro and/or anti-SSB/La antibodies) [17]. Secondary Sjögren’s syndrome (sSS) is defined as a disorder that occurs in association with another established autoimmune disorder, such as rheumatoid arthritis, systemic lupus erythematosus, or scleroderma [18,19]. Patients with secondary Sjögren’s syndrome may have additional features of the underlying disorder in addition to the symptoms of xerophthalmia and xerostomia [19].

Patients with Sjögren’s syndrome may experience a range of oral health complications as a result of xerostomia (dry mouth) caused by the disorder. These complications include [20,21,22]:

- Dental caries: xerostomia can increase the risk of dental caries due to the decreased salivary flow, which results in a decreased ability to neutralize acid and wash away bacteria and food particles.
- Oral candidosis: xerostomia can lead to an overgrowth of the fungus Candida, which can cause white, sore patches in the oral cavity, known as oral candidosis.
- Mucositis: Sjögren’s syndrome patients may also have an increased risk of oral mucositis (inflammation and ulceration of the mucous membranes).
- Dysgeusia (distorted sense of taste) and dysphagia (difficulty swallowing).

Effective oral hygiene practices, including regular dental check-ups, professional cleanings, and self-care, such as regular tooth brushing and flossing, are essential to mitigate these oral health complications [23]. Saliva substitutes and xerostomia-relieving medications may also be prescribed by dentists or physicians to alleviate symptoms. Patients with Sjögren’s syndrome should be aware of their oral symptoms and seek dental care as soon as possible if they experience any oral health complications [24].

Considering this background, the aim of this systematic review was to assess the existing literature on the connections between periodontal conditions and SS, on the premise that patients with SS may be more susceptible to periodontitis’ onset as compared to non-SS individuals.

## 2. Materials and Methods

**Figure 1:**
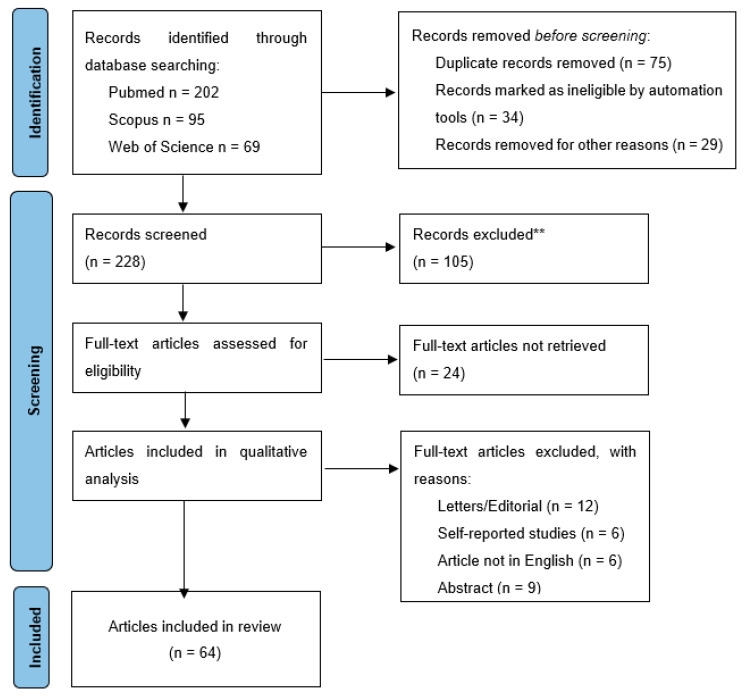
PRISMA flow diagram. (**—excluded according to exclusion criteria).

The Preferred Reporting Items for Systematic Review and Meta-Analyses (PRISMA) guidelines and regulations were implemented for the development and writing of this review (Figure 1). 

### 2.1. PICO Question

“Are patients with Sjogren’s syndrome (P) more susceptible to the onset of periodontitis (I) than the general population (C) and what therapeutical approach should be taken for these patients (O)?” (Population: patients with Sjögren’s Syndrome; intervention: susceptibility for periodontitis; comparison: general population, without Sjögren’s Syndrome; outcome: personalized periodontal approach for SS patients, in case of increased susceptibility).

### 2.2. Search Strategy

The database search was performed by a single researcher from October to December 2022, expanding to a timeline from 1964 to 2022. Relevant scientific databases which were searched included the following: Medline (via PubMed), Web of Science, and Scopus. The keywords used during this search were as follows: “Sjögren’s syndrome”, “periodontitis”, “sicca”, “dry mouth”, “periodontal status”, and “periodontal disease”, with Boolean operators “AND” and “OR” (Table 1).

### 2.3. Inclusion and Exclusion Criteria for the Selected Studies

The included studies had to meet the following criteria: in extenso publishing, full-text availability, and being written in English language. The exclusion criteria for the studies generated by the research were as follows: self-reported studies, letters and editorials, and abstracts. The selected studies for inclusion were submitted for further assessment, saved in Zotero software, and duplicates were removed.

### 2.4. Data Extraction

The information was carefully extracted from the papers by a single researcher, assessing the type of study, type of assessed parameters, and type of used biological samples, outputs, and conclusions. The data were compiled in a separate Microsoft Excel spreadsheet.

### 2.5. Information Structuring and Review Writing

The extracted information was divided into two main sections: fundamental and clinical research. Furthermore, the gathered data were subdivided considering the variously used parameters for a better comparison between similar types of results and a better understanding of the following relevant information: 1. epidemiological research data; 2. fundamental research data; 3. clinical research data.

## 3. Results and Discussion

### 3.1. Epidemiological Research Data

SS patients could be at a 2.2 times higher risk for developing periodontal disease [25]. The evaluated patients in the study group by Najera et al. exhibited significantly higher dental plaque (PLQ), clinical attachment loss (CAL), and bone loss compared to controls. Nevertheless, the number of cases between the study and control groups did not significantly differ. Soto-Rojas et al. [26] also estimated the risk of periodontitis in SS patients to be 2.2 times higher than in controls. Although the occurrence of periodontitis is frequent in these patients, the authors believe that the predominance of cariogenic bacteria instead of perio-pathogens leads to the absence of significant differences in the periodontal status of patients with SS and controls [26].

Patients with SS could have an increased risk of dental caries and periodontal diseases, such as gingivitis and periodontitis [27]. This study, with an adjusted incidence rate ratio of 1.43 for gingivitis and 1.44 for periodontitis on a large sample of 709 SS patients, was conducted as a national cohort study in Taiwan from 2000 to 2012. Given the large scale of the study, a justified raised awareness is advised, for rheumatologists and dental practitioners alike, for the increased risk of SS patients for developing dental and periodontal conditions [27].

The study by Leung et al. [28] evaluated the prevalence of periodontitis symptoms in 51 patients with SS. Despite 68% of the patients exhibiting calculus deposits, 48% gingival bleeding, and 80% periodontal pockets, the differences to the control group were not statistically significant. This led the authors to conclude that SS patients do not appear to be at a greater risk for periodontitis. In another study, periodontitis also exhibited similar prevalence among SS patients and controls [29], the gingival and periodontal status of the two groups not being significantly different, leading the authors to conclude that the risk of periodontitis was not increased in the SS population.

An epidemiological study [30] found a high prevalence of periodontal disease in children with SS (40%); however, the sample group was small (15 cases), thus limiting the relevance of the results. In a similar study [31], 90% of the evaluated SS patients exhibited signs of periodontal inflammation, the most frequent being bleeding on probing (93%) and spontaneous bleeding (24%). The relevance of gingival bleeding for the symptomatology of periodontal disease is high, as vascular gingival changes occur early in the development of low-grade local inflammation. The study by Boutsi et al. [32] showed that 75% of the assessed SS patients exhibited periodontal pockets upon periodontal examination, and 66.7% of them had a CAL between 4 and 6 mms. Despite these findings, the results were not significantly different from that of the control groups, leading the authors to conclude that the periodontal status of SS patients was similar to that of the general population. The authors speculate that the reduced salivary flow may affect the cervical areas of teeth more prominently, rather than the subgingival sites [32].

If the influence of SS on the pathogenesis and increased risk for periodontitis has been widely studied, conversely, it was also shown that patients with periodontitis could manifest a higher risk of subsequent SS development (50% higher than the general population), recommending that periodontists pay increased attention to the signs and symptoms of SS in patients with periodontal disease [33]. The authors also suggest that this pathogenic link may be mediated by a modified immune inflammatory response, contributing to this association [33]. Similar results were generated by the study of Lu et al. [34], showing that three years prior to SS diagnosis, the utilization of ambulatory dental services for gingivitis and periodontitis was significantly higher in these patients. Hence, dental practitioners could play an essential role in the early detection and diagnosis of primary SS [35].

The epidemiological data on the subject (Table 2) reflect that the risk for periodontitis could be increased in SS patients, although the opinion is not homogenous among the authors researching this topic.

### 3.2. Fundamental Research Data

Periodontitis is an inflammatory disease, its low-grade inflammation generated by the infiltration of subgingival bacteria being driven by a vast array of pro-inflammatory mediators [7]. Some of these cytokines have also been detected in increased levels in saliva samples of SS patients, including interleukin (IL)-12, T-cell activation factor (CD44), and β-2 macroglobulin [36]. This could suggest that the same up-regulated cytokines could be used for the development of low-grade periodontal inflammation [36]. Similar results were generated by a study by Moreno et al. [37] on salivary levels of IL-6 in saliva samples of pSS patients. These levels were significantly elevated and associated with pSS status, suggesting that this cytokine could be used as a biomarker for the diagnosis and monitoring of SS and its evolution. IL-6 is also a major pro-inflammatory marker for the pathogenesis of periodontitis, with a significant impact on bone resorption and osteoclast activation [37].

The involvement of key pro-inflammatory mediators in the pathogenesis of SS in connection with periodontitis was also assessed, showing the contribution of IL-17 [38]. The levels of this cytokine were elevated in plasma samples of SS patients, as well as the serum levels of IL-1beta, tumor growth factor (TGF), IL-6, and IL-23. These cytokines have also been shown to play important roles in the onset and development of the low-grade periodontal inflammatory reaction. The association between IL-6 levels and the periodontal status in SS patients showed that this cytokine exhibited up-regulated levels in serum and saliva samples of affected participants [39]. As IL-6 plays an important role in the inflammatory cascade reaction as an end-stage effector cytokine, the connection between common pathogenic pathways in SS and periodontitis could be influenced by this pro-inflammatory mediator [39]. Other key elements of the periodontal inflammatory reaction and its consequences on tissue dissolution, such as matrix-metalloproteinases (MMP)-2 and MMP-9, have been observed to be involved in the pathogenic processes of SS in animal models [40]. Moreover, patients with primary SS have been shown to exhibit variants of tumor necrosis factor (TNF)-alpha, a key mediator in the periodontal low-grade inflammatory reaction [39].

The expression of other relevant pro-inflammatory markers in gingival crevicular fluid (GCF) samples of SS patients has also been evaluated by the case–control study of Özçaka et al. [41]. While the GCF levels of caspase (CASP)-1 were significantly elevated in samples originating from SS patients, the levels of TNF-alpha and interferon (IFN) were not significantly different from those of controls. Nevertheless, when assessing IL-1beta in serum samples, the highest levels of this cytokine were found in SS patients, followed by rheumatoid arthritis (RA) patients, and, lastly, controls. In RA patients, the link between periodontitis and joint destruction was studied by Marotte et al. [42], illustrating an association between the severity of alveolar bone destruction and involvement of the human leukocyte antigen (HLA-DR) epitope in the inflammatory articular processes. The shared epitope was another severity genetic marker proposed by the authors for wrist joint and periodontal bone destruction [42]. Thus, the involvement of the autoimmune processes could be significant in the connection of periodontitis and conditions such as RA and SS, sometimes mediated by anti–Sjögren’s syndrome-related antigen A and B autoantibodies (anti-SSA/SSB positivity) in serum samples of affected patients [43].

Regarding enzymatic activity in periodontal inflammation, it was highlighted that collagenase and elastase levels were significantly lower in GCF samples of SS patients and controls compared to patients with periodontitis [44]. This suggests that periodontal pocket inflammatory activity is not affected by the involvement of glandular salivary tissue and that SS patients’ periodontal status was comparable to that of healthy patients [44]. Certain elements have been proposed as specific markers for the monitoring of the onset, development, and treatment efficiency for periodontitis and SS, as in the case of lipid mediators [45]. As shown by Shikama et al. [46], these include free fatty acids (FFA), with involvement in the pathogenic processes of both periodontitis and SS.

The immune reaction occurring in periodontitis and autoimmune diseases was also studied from a cellular point of view [47]. A study by Pers et al. described the correlation between the periodontal status of SS patients with their salivary B-cell activating factor levels [47]. The authors found that similar to patients with RA, the participants with periodontitis and SS exhibited higher serum and salivary B-cell activating factor (BAFF) levels than controls with periodontitis and no SS. This endorses the pathophysiologic link between periodontitis and SS, mediated by up-regulated BAFF levels and their positive correlation with PLQ, gingival index (GI), and bleeding on probing index (BOP) levels. Thus, the authors believe that B-cells could contribute to negative periodontal effects by up-regulation of the BAFF in the saliva of primary SS patients [47].

From a bacterial standpoint, it was shown that patients with SS expressed oral dysbiosis, with increased biofilm levels of *Streptococcus* spp. and *Veilonella* spp. [48]. Although this type of bacteria is mainly involved in the pathogenic process of tooth decay, they represent early biofilm colonizers, which create favorable environmental conditions for the settling of late bacterial colonizers, which are responsible for triggering the periodontal inflammatory reaction [2]. Thus, if left undisturbed by good oral hygiene practices, the dental biofilm in SS patients can easily shift to a periodontal pathogenic mode. This hypothesis is also supported by Lungoja et al. [49], reflecting elevated antibody levels against *P. denticola* in patients with primary SS. The authors also suggest the need for a more comprehensive analysis of the oral microbiome in SS patients, in order to identify potential bacterial triggers for this condition in relation to special bacteria involved in tooth decay or periodontitis pathogenesis [49].

In SS patients, antibody levels against *S. oralis* were significantly lower, while antibodies against *A. actinomycetemcomitans* and *P. gingivalis* (two major perio-pathogens) were significantly higher than in controls [50]. These immunological parameters to the bacterial challenge were correlated to the modified periodontal parameters in SS patients (PLQ, BOP, and periodontal pocket depth—PPD), which were significantly increased than in controls. This led the authors to diagnose SS patients with periodontitis in connection with increased antibody activity to periodontal pathogens, concluding that SS may affect biofilm bacterial colonization and contribute to an increased risk for periodontal disease [50]. Another study by the same authors [51] also adopted an interesting approach to the subject, evaluating histological changes occurring within the gingival epithelium of SS patients. The authors found the proliferative activity of cell nuclear antigen (PCNA) in the suprabasal layers of the gingival epithelium more frequently than in the basal layers. The authors suggest that as gingival inflammation leads to increased proliferative activity, this positive effect is down-regulated in SS patients due to their lower salivary flow. Thus, the biological mediators found within saliva could also contribute to the increased proliferative activity, offering protection against the challenge of perio-pathogenic bacteria [51].

Animal models were used in an experimental study assessing the role of periodontal disease in SS onset [52]. According to the study, the role of micro-RNA in modulating the onset of SS could offer a plausible mechanism for connections with periodontitis, as supported by observed changes in micro-RNA of salivary glands in rats with periodontitis. This could lead to the translocation of pathogenic bacteria and to an intricate association between periodontitis and SS. This association could be mediated by the observation of *P. gingivalis* and *T. denticola* in rat salivary glands, as well as by the decrease in micro-RNA in their submandibular salivary glands [52].

An interesting and novel approach to the association of SS and periodontitis was that of Scardina et al. [53], who assessed vascular changes occurring in the gingival tissue of SS patients. This study highlighted evident alterations to the gingival vascular capillary architecture and typical conformations of the interdental papillary microcirculation patterns. The observed changes in the reduced caliber of the papillary capillaries and increased complexity and quantity of capillary loops could suggest a decrease in gingival blood supply in SS patients. This, in return, could result in reduced trophicity and immune capabilities of the gingival connective tissue, that in the case of subgingival bacterial aggression, translates to increased susceptibility to periodontal disease [53].

A recent study suggests that dental care and therapy led to an improvement in salivary flow in pSS patients, both in unstimulated and stimulated scenarios, highlighting the need for close dental and periodontal monitoring of them [54]. This suggestion is also endorsed by the study of Singh et al. [55], showing that saliva is a major controlling factor for the development of intraoral biofilm. Thus, some bacterial species, mainly *V. parvula*, are closely associated with saliva flow, even being considered as a unique microbial biomarker for SS. This fact is of particular interest from a periodontal point of view, as this bacterium is considered to be a key periodontal pathogen [55].

However, some treatment options for SS had no improving effects on the periodontal status of SS patients. These trials include the use of pilocarpine, which improved stimulated and unstimulated saliva flow in pSS patients, but had no influence on the assessed periodontal parameters (PLQ, GI, and CAL) [56]. Various treatment options have been suggested for SS, but with limited results, as currently, the disease is considered to be untreatable. The use of pilocarpine has also been evaluated by Hsu et al. [57], showing that it offered no protective effect on the development of dental caries in patients with SS, periodontal disease, or oral candidosis, thus having little improvement in their oral health status, although increasing saliva flow.

The fundamental research (Table 3) performed on the topic of pathogenic connections between SS and periodontitis proposes varied and intriguing mechanisms that may mediate this link. However, so far, no definitive pathogenic mechanism can be attributed to a major role.

### 3.3. Clinical Research Data

The hypothesis of increased risk of periodontitis in SS patients is suggested by the meta-analysis of de Goes-Soares et al. [58], performed on 518 SS patients and 544 controls. The results of the meta-analysis showed that mean PLQ, GI, and BOP were higher in SS patients than in controls. The authors also recommended a closer oral examination of patients complaining of eye and mouth dryness for an early diagnosis of SS. Even though plaque accumulation was shown to be higher in SS patients, the authors could not conclude on strong evidence to demonstrate that periodontal status is affected by SS, due to substantial heterogeneity of the analyzed data [58]. Thus, the authors recommended further observational studies on the subject and advised careful monitoring of the periodontal status of SS patients in order to intercept early signs of periodontitis [58]. SS patients should be encouraged and offered support by dental practitioners to not only seek dental treatments, but also to maintain good oral hygiene, as PLQ and GI values have been shown to decrease in SS individuals that have already addressed dental offices for dental care [59].

The negative impact of SS on the periodontal status of affected patients was highlighted by Antoniazzi et al. [60]. In this study, SS patients exhibited significantly higher PLQ, GI, and CAL levels than controls. Even after adjusting the values for PLQ, the GI still kept significantly higher in the study group. The study also found differences between the periodontal status of pSS and sSS patients, in that sSS-type patients had significantly higher BOP, CAL, and PPD than their pSS counterparts. Thus, the authors concluded that, mainly in sSS patients, gingival inflammation is more prevalent and evident, and that a clear negative influence of SS could be observed on the periodontal status of affected patients. This could be a direct consequence of the decreased salivary flow, as in patients with hyposalivation, but with no SS, similar changes have been observed (increased BOP, PLQ) [61].

A recent meta-analysis [62] performed on 198 patients with SS highlighted higher PLQ and GI in these patients than in controls. However, the differences between PPD and CAL were not statistically significant. Thus, the authors concluded that the current results are inconclusive and stressed the need for future, well-designed observational studies on this topic in order to generate clearer outputs [62].

The study of Le Gall et al. [63] found higher PLQ, GI, and PBI levels in SS patients than in controls. Despite no significant difference in PPD, the authors state that the probability of severe periodontal conditions is more increased in SS patients than in controls due to their impaired salivary gland function and reduced saliva buffer capacity. Similar results were generated by other studies [64], considering that SS patients may have a higher risk of periodontitis or oral candidosis onset, as statistically significant differences were found between SS patients and controls for saliva flow, PLQ, BOP, and Candida albicans colonization values [65]. The differences between pSS and sSS were not significant, suggesting little impact on SS’s clinical form.

Nevertheless, the opinion of a less favorable periodontal status in SS is not homogenous among authors [66], who state that no consistent relationship could be found between the amount of saliva and the onset of periodontitis, as no increase in incidence and severity of periodontitis was observed in pSS patients. Pedersen et al. [67] also found no substantially elevated risk of periodontitis in pSS patients, as the authors could not identify any significant differences in periodontal conditions between SS patients and controls (no differences in PLQ and PPD, and no correlations with salivary flow rates). Rhodus et al. [68] also evaluated periodontal parameters in SS patients, and despite similar results on PPD and PLQ, their patients had significantly higher CAL values than controls. Furthermore, the GCF flow was increased in SS patients, suggesting the existence of an ongoing sub-clinical gingival inflammatory reaction and an increase in gingival recession risk [68].

Jorkjund et al. [69] concluded that SS patients do not appear to be at increased risk for periodontitis onset, but that periodontitis can occur in this category of patients as easily as it does in the general population. These conclusions were drawn on the premise that no statistical differences were observed in the periodontal status of SS patients and controls. Nevertheless, the authors did find more CAL in the mandible area of SS patients compared to controls, emphasizing that gingival recession is more frequent in these patients, probably in connection with reduced saliva flow and gingival connective fibers’ resistance to occlusal forces [69]. Similar conclusions were reached by Kuru et al. [70], stating that the periodontal status did not differ significantly between SS patients and controls, neither from a clinical (plaque deposits), inflammatory (peptidase activity), or microbiological perspective (type of periodontal microorganisms). Thus, the occurrence rate, severity, and extent of periodontal lesions were not significantly different between the test and control groups [71,72], even though SS leads to more systemic disease, medication intake, and poorer general health in most affected patients [72]. Taking these into consideration, it can be speculated that, even though at the baseline, SS patients did not appear to have an increased risk for periodontitis and no correlation was found between any of the salivary flow rates and periodontal parameters [73], the accumulation of SS-derived systemic diseases could, indirectly, elevate the predisposition of periodontitis in these patients.

Despite conflicting evidence in the existing literature, Maarse et al. [74] conclude that there was no evidence for a higher risk for periodontitis onset in patients with SS, due to no significant differences in the assessed GI, PLQ, CAL, and PPD parameters, as compared to healthy controls. Similar results were generated by other studies [75], concluding that SS has no observable influence on periodontal parameters. The authors stated that even if individuals with SS have a higher risk for tooth decay development, the risk for periodontal disease is insignificant. This statement could be argued that in itself, tooth decay represents an additional risk factor for the onset of periodontal conditions, mainly gingivitis, when manifesting near the gingival margin; therefore, the authors’ conclusion could be considered conflicting [75].

The conflicting nature of the results on the increased susceptibility for periodontitis in SS patients is also highlighted by other reviews and clinical studies on the subject [76]. In this paper, despite the fact that SS patients exhibited higher PLQ, GI, BOP, and decayed, missing, and filled teeth (DMFT) indexes than controls, the authors concluded that SS was unlikely to contribute to an increased risk of periodontitis in affected individuals. Nevertheless, the authors admit the limitations of their results due to the small sample of selected patients [76].

The treatment of edentulism in SS patients by dental implants was also evaluated [77]. The authors found that while the bone level around the dental implants was stable and the reduced salivary flow seemed not to affect the implant’s function, the overall implant success rate in SS patients was still slightly lower than in controls. Consequently, the authors encourage the use of dental implants in SS patients, as implant-supported prostheses offer good clinical advantages and significant life quality improvement [77]. These findings are also supported by Guobis et al. [78], showing that the early complication rate in 140 implants in SS patients was at only 3%, represented by mild mucositis, with little clinical relevance. After a 4-year recall, the prevalence of periimplantitis was 11%, similar to that of controls. Despite some studies reporting major peri-implant soft tissue alterations and increased BOP [79], implant therapy is advisable in SS patients, the majority being satisfied with results and benefits to everyday life [80].

Ambrosio et al. [81] studied the efficiency and results of non-surgical periodontal therapy (NSPT) in patients with SS and periodontitis. At baseline and after NSPT, the periodontal status was similar in SS patients and controls from a clinical, immunological, and microbiological perspective. Furthermore, NSPT proved to deliver significant improvements in salivary flow and Sjögren’s syndrome patient reported index (EULAR) value, suggesting that periodontal therapy may have a positive effect on the quality of life of SS patients dealing with periodontal disease. A similar study [82] delivered comparable results, highlighting an improvement in salivary flow after NSPT associated with the oral use of sodium bicarbonate and xylitol spray. Thus, the authors suggest the association of oral xylitol sprays in SS patients undergoing NSPT as an adjunctive mean to improve periodontal conditions and alleviate oral symptoms, such as pain and discomfort generated by oral mucosal dryness. The symptomatic treatment of SS is also supported by the study of Jadhav et al. [83] as an efficient method to prevent the development of tooth decay and periodontitis in affected patients. Combined with proper dental care, this approach may prevent the onset of periodontitis in SS patients and have a significant impact on their general health and well-being [84,85].

Concerning the association between periodontitis and other autoimmune diseases, it has been shown that this type of condition can significantly influence the manifestation of periodontal disease in terms of susceptibility and severity. This is the case for systemic sclerosis [86], where patients exhibited higher levels of periodontal inflammation and greater radiographic bone loss and RA, especially in the case of newly diagnosed cases [87].

The evaluation of the periodontal and oral status of SS patients, as depicted by the existing literature (Table 4), revealed conflicting results, with a high variability of the drawn conclusions. This is probably due to the increased heterogeneity of the study’s designs.

## 4. Future Perspectives

As highlighted by a recent meta-analysis on the subject, performed on 21 studies with a total of 11,435 participants, SS patients could have an increased risk of being diagnosed with periodontitis [88]. However, as reflected by our systematic review and the conclusion of Yang’s meta-analysis, the results are highly heterogenous, meaning that so far, no definitive conclusion could be drawn [88]. Our review has illustrated a variety of approaches to the matter of increased risk in SS patients for periodontitis, generating, at times, conflicting results. This motivates the future extension of research on this matter with well-designed, inclusive, longitudinal, or randomized clinical trials that would bring more conclusive results.

To explore the distinctive features of oral and periodontal health in SS patients, it may be necessary to conduct pilot trials on smaller patient groups. Initially, the project could involve a comparison of clinical data between individuals diagnosed with SS and controls, assessing the severity and progression rates of oral and periodontal health, including factors such as the number of missing teeth, periodontal diagnosis, and specific diagnosed periodontal conditions.

The study design and group characteristics would need to be established to include patients without coexisting systemic diseases, such as diabetes mellitus, that may affect the development of periodontitis. To measure pro-inflammatory mediators in GCF samples relevant to both periodontitis and Sjögren’s pathogenesis, an immunological analysis via the enzyme-linked immunosorbent assay method would be required. Additionally, clinical and immunological evaluations of periodontal therapy in patients diagnosed with periodontitis and Sjögren’s would be needed to assess the local and systemic effects of treatment and detect improvements in the expression of inflammatory mediators as an indicator of inflammatory reaction modulation.

## 5. Conclusions

The current scientific literature provides some degree of evidence of an increased risk of periodontitis onset in Sjögren’s syndrome patients, but the opinion is not homogenous among the authors researching this topic. This motivates the development of future studies on the subject, which would also bring new insights into the mechanisms mediating this possible connection.

## Figures and Tables

**Table 1:** Terms used in the research.

Database	Keyword Search
PUBMED	(“Sjögren, syndrome”[Mesh]) AND “Periodontitis”[Mesh]; (“Sjögren, syndrome”[Mesh]) AND “Periodontal Diseases”[Mesh]; (“Sjögren, syndrome”[Mesh]) AND “Periodontal Status”[Mesh]; (“Sicca, syndrome”[Mesh]) AND “Periodontitis”[Mesh]; (“dry, mouth”[Mesh]) AND “Periodontitis”[Mesh];
Web of Science	TS = (“Sjögren syndrome”) AND TS = (“Periodontal Diseases”); TS = (“Sjögren syndrome”) AND TS = (“Periodontitis”); TS = (“dry mouth”) AND TS = (“Periodontitis”).
SCOPUS	ALL(“Sjögren syndrome”) AND ALL(“Periodontal Diseases”); ALL(“Sjögren syndrome”) AND ALL(“Periodontitis”); TITLE-ABS-KEY(“Sjögren syndrome”) AND TITLE-ABS-KEY(“Periodontal Diseases”); TITLE-ABS-KEY(“Sjögren syndrome”) AND TITLE-ABS-KEY(“Periodontitis”).

**Table 2:** Synopsis of the selected papers with epidemiological research data and their findings.

Reference	Type of Study	SS Group (No.)	Findings
Highlighted link between periodontitis and SS			
Al-Hashimi et al., 2001 [35]	Review	/	Dentists and dental hygienists can help identify early signs and symptoms of SS
Lin et al., 2019 [33]	Retrospective cohort analysis	3292	Patients with periodontitis had a significantly more elevated risk of subsequent SS onset; dental practitioners should be aware of SS risk
Lu et al., 2014 [34]	Retrospective cohort analysis	389	Dental and periodontal care is essential in early recognition of SS signs and symptoms
Chuang et al., 2020 [27]	Retrospective cohort analysis	709	Increased risk for periodontal disease onset in SS patients; rheumatologists should be vigilant for oral health of SS patients
Najera et al., 1997 [25]	Transversal	25	SS patients have a 2.2 times increased risk for developing periodontitis than controls
Olate et al., 2014 [31]	Transversal	35	Increased prevalence of periodontal inflammation (gingival bleeding) in SS patients
Conde et al., 2021 [30]	Retrospective	15	Increased prevalence of periodontitis in children with SS
No link between periodontitis and SS			
Soto-Rojas et al., 2002 [26]	Review	/	No significant difference in periodontal status of SS patients and controls
Leung et al., 2004 [28]	Transversal	51	SS patients appear not to be at increased risk for periodontal disease than controls; periodontal parameters cannot be used for SS detection
Schiødt et al., 2001 [29]	Transversal	57	Periodontal disease has similar prevalence among SS and control participants
Boutsi et al., 2000 [32]	Transversal	24	No significant difference in periodontal status in SS patients compared to control group

**Table 3:** Synopsis of the selected papers with fundamental research data and their findings.

Reference	Type of Study	SS Group (No.)	Findings
Highlighted link between periodontitis and SS			
Maciejczyk et al., 2016 [40]	Review	/	MMP-2 and MMP-9 involvement in SS pathogenesis
Bunte et al., 2019 [38]	Review	/	IL-17 has important role in the pathogenesis of SS and periodontitis
Sommakia et al., 2016 [45]	Review	/	Lipid mediators’ saliva levels could be an additional tool for study of SS and periodontitis etiology, development, and treatment
Shikama et al., 2017 [46]	Review	/	Common involvement of free fatty acids in the pathogenesis of periodontitis and SS
Nibali et al., 2012 [39]	Review	/	Association between SS and periodontitis, mediated by IL-6, end-stage effector cytokine in the inflammatory reaction
De Luca et al., 2019 [48]	Review	/	Increased levels of *Streptococcus* spp. and *Veillonella* spp. in SS patients
M. Rodriguez et al., 2020 [36]	Review	/	Increased levels of relevant pro-inflammatory mediators in saliva of SS patients
Martins et al., 2022 [54]	Prospective	52	Primary dental care promoted improvement in salivary flow rates in SS patients
Pers et al., 2005 [47]	Transversal	15	B-cells could contribute to periodontal complications through up-regulation of BAFF in saliva of SS patients
Lungoja et al., 2016 [49]	Transversal	39	Increased *P. denticola* antibody levels in SS patients, identification of potential bacterial triggers
Marton et al., 2006 [43]	Case–control	49	Oral health status of SS patients may be associated with the general autoimmune process (anti-SSA/SSB antibodies)
Moreno et al., 2020 [37]	Case–control	36	IL-6 expressed elevated levels in saliva of SS patients; this cytokine could be useful in the diagnosis and follow-up of SS patients
Celenligil et al., 1998 [50]	Case–control	17	SS may affect bacterial colonization in dental biofilm and contribute to elevation of periodontitis risk
Celenligil et al., 2003 [51]	Case–control	18	Inflammation causes increased proliferative activity, but this positive effect is downregulated by the reduced saliva flow in SS
Scardina et al., 2010 [53]	Case–control	25	Evident alterations to the capillaries and typical conformation of the interdental papilla microcirculation in SS patients
Singh et al., 2021 [55]	Case–control	57	Saliva is a major controlling factor for oral biofilm (impact of *V. parvula* in SS patients)
Nayar et al., 2016 [52]	Experimental	/	The role of micro-RNA in modulating the development of SS offers plausible mechanisms for association with periodontitis
No link between periodontitis and SS			
Ozcaka et al., 2018 [41]	Case–control	44	No significant difference in periodontal status and GCF levels of TNF-alpha and IFN-gamma between SS patients and controls
Tervahartiala et al., 1995 [44]	Comparative	8	Periodontal pockets development is not affected by involvement of glandular tissue (lower collagenase and elastase activity in SS)
Depinoy et al., 2021 [56]	Prospective cohort	19	Treatment with pilocarpine showed no improvement in periodontal parameters in SS patients
Hsu et al., 2019 [57]	Prospective cohort	487	Pilocarpine may have no protective effect on dental caries and periodontitis in SS patients

**Table 4:** Synopsis of the selected papers with clinical research data and their findings.

Reference	Type of Study	SS Group (No.)	Findings
Highlighted link between periodontitis and SS			
Chatzistavrianou et al., 2016 [77]	Review	/	The clinical advantages proved by implant prosthodontics outpace the slightly lower overall implant success rate in SS patients
Fox et al., 1986 [65]	Review	/	Patients with SS present specific therapeutic problems: increased oral infections and periodontitis
Albrecht et al., 2016 [80]	Observational cohort	205	Majority of SS patients were satisfied with dental implants and would recommend them to others
Rhodus et al., 2005 [68]	Case–control	10	SS patients have significantly more gingival recession and GCF flow than healthy controls
Ergun et al., 2010 [64]	Case–control	37	SS patients could have a higher risk for periodontitis
Gambino et al., 2017 [82]	Randomized control trial	22	Gingival status of SS patients improved after non-surgical periodontal therapy, as well as salivary flow
Lucchese et al., 2018 [59]	Clinical Trial	52	Significant decrease in biofilm deposits and gingival inflammation before and after dental care of SS patients
Johansson et al., 2001 [85]	Clinical trial	22	Observed positive effects on symptoms in patients with SS after use of chlorhexidine
Antoniazzi et al., 2009 [60]	Comparative	19	SS negatively affects the periodontal status and increased gingival inflammation in patients with SS
Pedersen et al., 2002 [72]	Comparative	20	SS predisposes to more systemic disease, increased medication intake, and poor general health, risk factors for periodontitis
Le Gall et al., 2016 [63]	Prospective	31	More severe periodontal conditions in SS patients than non-SS due to impaired salivary gland function and reduced buffer capacity
Marton et al., 2008 [61]	Transversal	/	Increased biofilm deposits and gingival bleeding in a hyposalivation scenario
Weinlander et al., 2010 [79]	Retrospective	5	Major peri-implant soft tissue alterations in patients with SS
Jadhav et al., 2015 [83]	Case report	/	Need for symptomatic treatment in SS patients to prevent periodontal disease
Lins et al., 2014 [84]	Case report	/	Appropriate dental care in SS patients may prevent periodontitis, improving the patient’s quality of life
No link between periodontitis and SS			
Wu et al., 2021 [62]	Meta-analysis	198	Clinical attachment loss and pocket depth were similar in SS patients and healthy controls; need for well-designed observational studies
Maarse et al., 2019 [74]	Meta-analysis	/	No evidence of a higher periodontitis risk in patients with SS
Bolstad et al., 2016 [76]	Review	/	SS does not seem to contribute to more periodontal disease than in healthy persons
Goubis et al., 2016 [78]	Review	/	Implant therapy is advisable and offers acceptable success rates in SS patients
Napenas et al., 2014 [66]	Review	/	No increase in incidence and severity of periodontitis in SS patients; no consistent relationship between saliva flow and periodontitis
De Goes-Soares et al., 2018 [58]	Review	518	No strong evidence that the periodontal status is influenced by SS
Ambrosio et al., 2017 [81]	Longitudinal prospective	28	SS patients have similar periodontal status to controls at baseline and after non-surgical periodontal therapy
Pedersen et al., 2005 [73]	Case–control	20	Presence of periodontal disease is not substantially increased in SS patients
Ravald et al., 1998 [71]	Case–control	21	The periodontal conditions are similar in SS patients to those found in the general population
Tseng et al., 1991 [75]	Comparative	14	SS has no observable influence on the assessed periodontal indexes
Kuru et al., 2002 [70]	Comparative	18	Periodontal status does not differ significantly between SS patients and age–gender-matched controls
Jorkjund et al., 2003 [69]	Case–control	33	SS patients are not specifically at risk of periodontitis

## Data Availability

Not applicable.

## Abbreviations

AECG	American–European Consensus Group	
Anti-SSA	anti-Sjögren’s syndrome-related antigen A autobodies	
BAFF	B-cell activating factor	
BOP	bleeding on probing index	
CAL	Gingival clinical attachment loss	
CASP	Caspase	
DMFT	Decayed, missing, and filled teeth	
EULAR	Sjögren’s syndrome patient reported index	
FFA	Free fatty acids	
GCF	Gingival crevicular fluid	
GI	Gingival index	
HLA	Human leukocite antigen	
IFN	Interferon	
IL	Interleukin	
MMP	Matrix-metalloproteinases	
NSPT	Non-surgical periodontal therapy	
PCNA	Proliferative activity of cell nuclear antigen	
PICO	Population Intervention Comparison Outcome	
PLQ	Dental plaque index	
PPD	Periodontal pocket depth	
PRISMA	Preferred Reporting Items for Systematic Review and Meta-Analyses	
pSS	Primary Sjögren’s syndrome	
RA	Rheumatoid arthritis	
SS	Sjögren’s syndrome	
sSS	Secondary Sjögren’s syndrome	
TGF	Tumor growth factor	
TNF	Tumor necrosis factor	

## References

[1] Kwon T.H., Lamster I.B., Levin L. (2021). Current concepts in the management of periodontitis. Int. Dent. J..

[2] Prado M.M., Figueiredo N., Pimenta A.D.L., Miranda T.S., Feres M., Figueiredo L.C., de Almeida J., Bueno-Silva B. (2022). Recent updates on microbial biofilms in periodontitis: An analysis of in vitro biofilm models. Periodontitis.

[3] Yucel-Lindberg T., Båge T. (2013). Inflammatory mediators in the pathogenesis of periodontitis. Expert Rev. Mol. Med..

[4] Herrera D., Sanz M., Kebschull M., Jepsen S., Sculean A., Berglundh T., Papapanou P.N., Chapple I., Tonetti M.S., Kopp I. (2022). Treatment of stage iv periodontitis: The EFP s3 level clinical practice guideline. J. Clin. Periodontol..

[5] Beck J.D., Papapanou P.N., Philips K.H., Offenbacher S. (2019). Periodontal medicine: 100 years of progress. J. Dent. Res..

[6] Fischer R.G., Gomes Filho I.S., Cruz S.S., Oliveira V.B., Lira-Junior R., Scannapieco F.A., Rego R.O. (2021). What Is the Future of Periodontal Medicine?. Braz. Oral Res..

[7] Caton J.G., Armitage G., Berglundh T., Chapple I.L.C., Jepsen S., Kornman K.S., Mealey B.L., Papapanou P.N., Sanz M., Tonetti M.S. (2018). A new classification scheme for periodontal and peri-implant diseases and conditions—Introduction and key changes from the 1999 classification. J. Clin. Periodontol..

[8] Loos B.G. (2016). Periodontal medicine: Work in progress!. J. Clin. Periodontol..

[9] Brito-Zerón P., Baldini C., Bootsma H., Bowman S.J., Jonsson R., Mariette X., Sivils K., Theander E., Tzioufas A., Ramos-Casals M. (2016). Sjögren syndrome. Nat. Rev. Dis. Prim..

[10] Baer A.N., Walitt B. (2018). Update on Sjögren syndrome and other causes of Sicca in older adults. Rheum. Dis. Clin. N. Am..

[11] Luppi F., Sebastiani M., Sverzellati N., Cavazza A., Salvarani C., Manfredi A. (2020). Lung complications of Sjögren syndrome. Eur. Respir. Rev..

[12] Margaretten M. (2017). Neurologic manifestations of primary sjögren syndrome. Rheum. Dis. Clin. N. Am..

[13] Theander E., Jacobsson L.T.H. (2008). Relationship of sjögren’s syndrome to other connective tissue and autoimmune disorders. Rheum. Dis. Clin. N. Am..

[14] Seror R., Nocturne G., Mariette X. (2021). Current and future therapies for primary Sjögren syndrome. Nat. Rev. Rheumatol..

[15] Akpek E.K., Bunya V.Y., Saldanha I.J. (2019). Sjögren’s syndrome: More than just dry eye. Cornea.

[16] Stefanski A.-L., Tomiak C., Pleyer U., Dietrich T., Burmester G.R., Dörner T. (2017). The diagnosis and treatment of Sjögren’s syndrome. Dtsch. Ärzteblatt Int..

[17] Jonsson R., Brokstad K.A., Jonsson M.V., Delaleu N., Skarstein K. (2018). Current concepts on Sjögren’s syndrome—Classification criteria and biomarkers. Eur. J. Oral Sci..

[18] Weerasinghe W.S., Jayasinghe C. (2022). Overlapping rheumatoid arthritis and antisynthetase syndrome with Secondary Sjögren’s syndrome: A case report and review of the literature. J. Med. Case Rep..

[19] Popov Y., Salomon-Escoto K. (2018). Gastrointestinal and hepatic disease in Sjögren syndrome. Rheum. Dis. Clin. N. Am..

[20] González S., Sung H., Sepúlveda D., González M.J., Molina C. (2013). Oral manifestations and their treatment in sjögren′s syndrome. Oral Dis..

[21] Choudhry H.S., Hosseini S., Choudhry H.S., Fatahzadeh M., Khianey R., Dastjerdi M.H. (2022). Updates in diagnostics, treatments, and correlations between oral and ocular manifestations of Sjögren’s syndrome. Ocul. Surf..

[22] Mathews S.A., Kurien B.T., Scofield R.H. (2008). Oral manifestations of Sjögren’s syndrome. J. Dent. Res..

[23] López-Pintor R.M., Fernández Castro M., Hernández G. (2015). Afectación oral en el paciente con síndrome de Sjögren Primario. Manejo multidisciplinar entre odontólogos Y reumatólogos. Reumatol. Clínica.

[24] Pinto A. (2014). Management of xerostomia and other complications of Sjögren’s syndrome. Oral Maxillofac. Surg. Clin. N. Am..

[25] Najera M.P., Al-Hashimi I., Plemons J.M., Rivera-Hidalgo F., Rees T.D., Haghighat N., Wright J.M. (1997). Prevalence of periodontal disease in patients with Sjögren’s syndrome. Oral Surg. Oral Med. Oral Pathol. Oral Radiol. Endodontology.

[26] Soto-Rojas A.E., Kraus A. (2002). The oral side of Sjögren syndrome. diagnosis and treatment. A Review. Arch. Med. Res..

[27] Chuang C.-J., Hsu C.-W., Lu M.-C., Koo M. (2020). Increased risk of developing dental diseases in patients with primary Sjögren’s syndrome—A secondary cohort analysis of population-based claims data. PLoS ONE.

[28] Leung K.C.M., McMillan A.S., Leung W.K., Wong M.C.M., Lau C.S., Mok T.M.Y. (2004). Oral health condition and saliva flow in southern Chinese with Sjögren’s syndrome. Int. Dent. J..

[29] Schiødt M., Christensen L.B., Petersen P.E., Thorn J.J. (2001). Periodontal disease in primary Sjögren’s syndrome. Oral Dis..

[30] Condé K., Guelngar C.O., Barry M.C., Atakla H.G., Mohamed A., Cissé F. (2021). Sjögren’s syndrome in children: About 15 cases in Guinea conakry. Eur. J. Med. Res..

[31] Olate S., Muñoz D., Neumann S., Pozzer L., Cavalieri-Pereira L., de Moraes M. (2014). A descriptive study of the oral status in subjects with Sjögren’s syndrome. Int. J. Clin. Exp. Med..

[32] Boutsi E.A., Paikos S., Dafni U.G., Moutsopoulos H.M., Skopouli F.N. (2000). Dental and periodontal status of Sjögren’s syndrome. J. Clin. Periodontol..

[33] Lin C.-Y., Tseng C.-F., Liu J.-M., Chuang H.-C., Lei W.-T., Liu L., Yu Y.-C., Hsu R.-J. (2019). Association between periodontal disease and subsequent Sjögren’s syndrome: A nationwide population-based Cohort Study. Int. J. Environ. Res. Public Health.

[34] Lu M.-C., Jheng C.-H., Tsai T.-Y., Koo M., Lai N.-S. (2014). Increased dental visits in patients prior to diagnosis of primary sjögren’s syndrome: A population-based study in Taiwan. Rheumatol. Int..

[35] Al-Hashimi I. (2001). Oral and periodontal status in Sjögren’s syndrome. Tex. Dent. J..

[36] Melguizo-Rodríguez L., Costela-Ruiz V.J., Manzano-Moreno F.J., Ruiz C., Illescas-Montes R. (2020). Salivary biomarkers and their application in the diagnosis and monitoring of the most common oral pathologies. Int. J. Mol. Sci..

[37] Moreno-Quispe L.A., Serrano J., Virto L., Sanz M., Ramírez L., Fernández-Castro M., Hernández G., López-Pintor R.M. (2020). Association of salivary inflammatory biomarkers with primary Sjögren’s syndrome. J. Oral Pathol. Med..

[38] Bunte K., Beikler T. (2019). Th17 cells and the il-23/il-17 axis in the pathogenesis of periodontitis and immune-mediated inflammatory diseases. Int. J. Mol. Sci..

[39] Nibali L., Fedele S., D’Aiuto F., Donos N. (2011). Interleukin-6 in oral diseases: A Review. Oral Dis..

[40] Maciejczyk M., Pietrzykowska A., Zalewska A., Knaś M., Daniszewska I. (2016). The significance of matrix metalloproteinases in oral diseases. Adv. Clin. Exp. Med..

[41] Özçaka Ö., Alpöz E., Nalbantsoy A., Karabulut G., Kabasakal Y. (2018). Clinical periodontal status and inflammatory cytokines in primary SJÖGREN syndrome and rheumatoid arthritis. J. Periodontol..

[42] Marotte H. (2005). The association between periodontal disease and joint destruction in rheumatoid arthritis extends the link between the HLA-DR shared epitope and severity of Bone Destruction. Ann. Rheum. Dis..

[43] Marton K., Boros I., Varga G., Zelles T., Fejerdy P., Zeher M., Nagy G. (2006). Evaluation of palatal saliva flow rate and oral manifestations in patients with Sjögren’s syndrome. Oral Dis..

[44] Tervahartiala T., Ingman T., Sorsa T., Ding Y., Kangaspunta P., Konttinen Y.T. (1995). Proteolytic enzymes as indicators of periodontal health in Gingival crevicular fluid of patients with Sjögren’s syndrome. Eur. J. Oral Sci..

[45] Sommakia S., Baker O.J. (2016). Regulation of inflammation by lipid mediators in oral diseases. Oral Dis..

[46] Shikama Y., Kudo Y., Ishimaru N., Funaki M. (2017). Potential role of free fatty acids in the pathogenesis of periodontitis and Primary Sjögren’s syndrome. Int. J. Mol. Sci..

[47] Pers J.-O., d’Arbonneau F., Devauchelle-Pensec V., Saraux A., Pennec Y.-L., Youinou P. (2005). Is periodontal disease mediated by salivary BAFF in Sjögren’s syndrome?. Arthritis Rheum..

[48] De Luca F., Shoenfeld Y. (2018). The microbiome in autoimmune diseases. Clin. Exp. Immunol..

[49] Lugonja B., Yeo L., Milward M.R., Smith D., Dietrich T., Chapple I.L., Rauz S., Williams G.P., Barone F., de Pablo P. (2016). Periodontitis prevalence and serum antibody reactivity to periodontal bacteria in primary Sjögren’s syndrome: A pilot study. J. Clin. Periodontol..

[50] Çelenligil H., Eratalay K., Kansu E., Ebersole J.L. (1998). Periodontal status and serum antibody responses to oral microorganisms in Sjögren’s syndrome. J. Periodontol..

[51] Çelenligil-Nazlıel H., Palalı A., Ayhan A., Ruacan Ş. (2003). Analysis of in situ proliferative activity in oral gingival epithelium in patients with xerostomia. J. Periodontol..

[52] Nayar G., Gauna A., Chukkapalli S., Velsko I., Kesavalu L., Cha S. (2016). Polymicrobial infection alter inflammatory microrna in rat salivary glands during periodontal disease. Anaerobe.

[53] Scardina G.A., Ruggieri A., Messina P. (2009). Periodontal disease and Sjögren Syndrome: A possible correlation?. Angiology.

[54] Martins V.A.O., Floriano T.F., Leon E.P., Villamarín L.E.B., Deveza G.B.H., Aikawa N.E., Silva C.A.A., Kupa L.V.K., Peres M.P., Braz-Silva P.H. (2022). Primary dental care treatment in primary Sjögren’s syndrome: A possible role in improving salivary flow rate. Clin. Exp. Rheumatol..

[55] Singh M., Teles F., Uzel N.G., Papas A. (2020). Characterizing microbiota from Sjögren’s syndrome patients. JDR Clin. Transl. Res..

[56] Depinoy T., Saraux A., Pers J.-O., Boisramé S., Cornec D., Marhadour T., Guellec D., Devauchelle-Pensec V., Bressollette L., Jousse-Joulin S. (2020). Salivary glands and periodontal changes in a population of Sjögren’s and Sicca syndrome treated by pilocarpine: A pilot study. Rheumatol. Ther..

[57] Hsu C.-Y., Hung K.-C., Lin M.-S., Ko C.-H., Lin Y.-S., Chen T.-H., Lin C.-Y., Chen Y.-C. (2019). The effect of Pilocarpine on dental caries in patients with primary Sjögren’s syndrome: A database prospective cohort study. Arthritis Res. Ther..

[58] De Goés Soares L., Rocha R.L., Bagordakis E., Galvão E.L., Douglas-de-Oliveira D.W., Falci S.G. (2018). Relationship between Sjögren syndrome and periodontal status: A systematic review. Oral Surg. Oral Med. Oral Pathol. Oral Radiol..

[59] Lucchese A., Portelli M., Marcolina M., Nocini P.F., Caldara G., Bertossi D., Lucchese C., Tacchino U., Manuelli M. (2018). Effect of dental care on the oral health of Sjögrens syndrome patients. J. Biol. Regul. Homeost. Agents.

[60] Antoniazzi R.P., Miranda L.A., Zanatta F.B., Islabão A.G., Gustafsson A., Chiapinotto G.A., Oppermann R.V. (2009). Periodontal conditions of individuals with Sjögren’s syndrome. J. Periodontol..

[61] Márton K., Madléna M., Bánóczy J., Varga G., Fejérdy P., Sreebny L.M., Nagy G. (2008). Unstimulated whole saliva flow rate in relation to sicca symptoms in Hungary. Oral Dis..

[62] Wu S.-Y., Wu C.-Y., Chen M.-H., Huang H.-Y., Chen Y.-h., Tsao Y.-P., Lai Y.-L., Lee S.-Y. (2021). Periodontal conditions in patients with Sjögren’s syndrome: A meta-analysis. J. Dent. Sci..

[63] Le Gall M., Cornec D., Pers J.-O., Saraux A., Jousse-Joulin S., Cochener B., Roguedas-Contios A.-M., Devauchelle-Pensec V., Boisramé S. (2016). A prospective evaluation of dental and periodontal status in patients with suspected Sjögren’s syndrome. Jt. Bone Spine.

[64] Ergun S., Cekici A., Topcuoglu N., Migliari D.A., Kulekci G., Tanyeri H., Isik G. (2010). Oral status and candida colonization in patients with Sjögren’s syndrome. Med. Oral Patol. Oral Y Cir. Bucal.

[65] Fox R., Howell F. (1961). Oral problems in patients with Sjögren’s syndrome. Scand. J. Rheumatol..

[66] Napeñas J.J., Rouleau T.S. (2014). Oral complications of Sjögren’s syndrome. Oral Maxillofac. Surg. Clin. N. Am..

[67] Pedersen A.M., Bardow A., Nauntofte B. (2005). Salivary changes and dental caries as potential oral markers of autoimmune salivary gland dysfunction in primary Sjögren’s syndrome. BMC Clin. Pathol..

[68] Rhodus N.L., Michalowicz B.S. (2005). Periodontal status and sulcular Candida albicans colonization in patients with primary Sjögren’s Syndrome. Quintessence Int..

[69] Jorkjend L., Johansson A., Johansson A.-K., Bergenholtz A. (2003). Periodontitis, caries and salivary factors in Sjögren’s syndrome patients compared to sex- and age-matched controls. J. Oral Rehabil..

[70] Kuru B., McCullough M.J., Yilmaz S., Porter S.R. (2002). Clinical and microbiological studies of periodontal disease in Sjögren’s syndrome patients. J. Clin. Periodontol..

[71] Ravald N., List T. (1998). Caries and periodontal conditions in patients with primary Sjögren’s syndrome. Swed. Dent. J..

[72] Pedersen A., Andersen Torpet L., Reibel J., Holmstrup P., Nauntofte B. (2002). Oral findings in patients with primary Sjögren’s syndrome and oral lichen planus—A preliminary study on the effects of bovine colostrum-containing oral hygiene products. Clin. Oral Investig..

[73] Pedersen A.M., Reibel J., Nordgarden H., Bergem H.O., Jensen J.L., Nauntofte B. (2008). Primary sjögren’s syndrome: Salivary gland function and clinical oral findings. Oral Dis..

[74] Maarse F., Jager D.H.J., Alterch S., Korfage A., Forouzanfar T., Vissink A., Brand H.S. (2019). Sjögren’s syndrome is not a risk factor for periodontal disease: A systematic review. Clin. Exp. Rheumatol..

[75] Tseng C.C. (1990). Periodontal status of patients with Sjögren’s syndrome: A cross-sectional study. J. Formos. Med. Assoc. Taiwan Yi Zhi.

[76] Bolstad A.I., Skarstein K. (2016). Epidemiology of sjögren’s syndrome—From an oral perspective. Curr. Oral Health Rep..

[77] Chatzistavrianou D., Shahdad S. (2016). Implant Treatment in Patients with Sjögren’s Syndrome: A Review of the Literature and Two Clinical Case Reports. Eur. J. Prosthodont. Restor. Dent..

[78] Guobis Z., Pacauskiene I., Astramskaite I. (2016). General diseases influence on Peri-Implantitis Development: A Systematic Review. J. Oral Maxillofac. Res..

[79] Weinlander M., Krennmair G., Piehslinger E. (2010). Implant prosthodontic rehabilitation of patients with rheumatic disorders: A case series report. Int. J. Prosthodont..

[80] Albrecht K., Callhoff J., Westhoff G., Dietrich T., Dörner T., Zink A. (2016). The prevalence of dental implants and related factors in patients with Sjögren Syndrome: Results from a cohort study. J. Rheumatol..

[81] Ambrósio L.M., Rovai E.D.S., França B.N., Balzarini D.A., Abreu I.S., Lopes S.B., Nunes T.B., Lourenço S.V., Pasoto S.G., Saraiva L. (2017). Effects of periodontal treatment on primary SJȪGREN’s syndrome symptoms. Braz. Oral Res..

[82] Gambino A., Broccoletti R., Cafaro A., Cabras M., Carcieri P., Arduino P.G. (2016). Impact of a sodium carbonate spray combined with professional oral hygiene procedures in patients with Sjögren’s syndrome: An explorative study. Gerodontology.

[83] Jadhav S., Jadhav A., Thopte S., Marathe S., Vhathakar P., Chivte P., Jamkhande A. (2015). Sjögren’s Syndrome: A Case Study. J. Int. Oral Health JIOH.

[84] Lins L., Paraná R., Reis S.R., Pereira Falcão A.F. (2014). Primary biliary cirrhosis and primary Sjögren’s syndrome: Insights for the stomatologist. Case Rep. Gastroenterol..

[85] Johansson G., Andersson G., Edwardsson S., Bjorn A.-L., Manthorpe R., Attstrom R. (2001). Effects of mouthrinses with linseed extract salinumr without/with chlorhexidine on oral conditions in patients with Sjögren’s syndrome. A double-blind crossover investigation. Gerodontology.

[86] Leung W.K., Chu C.H., Mok M.Y., Yeung K.W.S., Ng S.K.S. (2011). Periodontal status of adults with systemic sclerosis: Case-control study. J. Periodontol..

[87] Chen H.-H., Huang N., Chen Y.-M., Chen T.-J., Chou P., Lee Y.-L., Chou Y.-J., Lan J.-L., Lai K.-L., Lin C.-H. (2012). Association between a history of periodontitis and the risk of rheumatoid arthritis: A nationwide, population-based, Case–Control Study. Ann. Rheum. Dis..

[88] Yang B., Pang X., Guan J., Liu X., Li X., Wang Y., Chen Z., Cheng B. (2023). The Association of Periodontal Diseases and Sjögren’s syndrome: A systematic review and meta-analysis. Front. Med..

